# Ambivalent role of pFAK-Y397 in serous ovarian cancer-a study of the OVCAD consortium

**DOI:** 10.1186/1476-4598-13-67

**Published:** 2014-03-21

**Authors:** Stefanie Aust, Katharina Auer, Anna Bachmayr-Heyda, Carsten Denkert, Jalid Sehouli, Ioana Braicu, Sven Mahner, Sandrina Lambrechts, Ignace Vergote, Christoph Grimm, Reinhard Horvat, Dan Cacsire Castillo-Tong, Robert Zeillinger, Dietmar Pils

**Affiliations:** 1 Department of Obstetrics and Gynecology, Molecular Oncology Group, Comprehensive Cancer Center, Medical University of Vienna, Waehringer Guertel 18-20, Room-No.: 5.Q9.27, A-1090 Vienna, Austria; 2 Department of Gynecology, Campus Virchow Klinikum, Charite Medical University, European Union, Berlin, Germany; 3 Department of Gynecology and Gynecologic Oncology, University Medical Center Hamburg-Eppendorf, European Union, Hamburg, Germany; 4 Department of Obstetrics and Gynecology, Division of Gynecological Oncology, University Hospitals Leuven, Katholieke Universiteit Leuven, European Union, Leuven, Belgium; 5 Clinical Institute of Pathology, Medical University of Vienna, European Union, Vienna, Austria

**Keywords:** FAK, pFAK, Molecular subclassification, Immunohistochemistry, Prognosis, Ovarian cancer

## Abstract

**Background:**

Focal adhesion kinase (FAK) autophosphorylation seems to be a potential therapeutic target but little is known about the role and prognostic value of FAK and pFAK in epithelial ovarian cancer (EOC). Recently, we validated a gene signature classifying EOC patients into two subclasses and revealing genes of the focal adhesion pathway as significantly deregulated.

**Methods:**

FAK expression and pFAK-Y397 abundance were elucidated by immunohistochemistry and microarray analysis in 179 serous EOC patients. In particular the prognostic value of phosphorylated FAK (pFAK-Y397) and FAK in advanced stage EOC was investigated.

**Results:**

Multiple Cox-regression analysis showed that high pFAK abundance was associated with improved overall survival (HR 0.54; p = 0.034). FAK was positive in a total of 92.2% (n = 165) and high pFAK abundance was found in 36.9% (n = 66). High pFAK abundance (36.9% ; n = 66) was associated with either nodal positivity and/or distant metastasis (p = 0.030). Whole genome gene expression data revealed a connection of the FAK-pFAK-Y397 axis and the mTOR-S6K1 pathway, shown to play a major role in carcinogenesis.

**Conclusion:**

The role of pFAK-Y397 remains controversial: although high pFAK-Y397 abundance is associated with distant and lymph node metastases, it is independently associated with improved overall survival.

## Background

Despite increasing knowledge of the etiology and treatment of epithelial ovarian cancer (EOC), ovarian cancer still has the highest death rate compared to any other gynecological malignancy [1]. Trying to understand the biology of ovarian cancer, gene expression analysis and molecular subclassification is a challenging, but important strategy [2,3].

So far, no molecular signature for ovarian cancer is used in a routine clinical setting to classify patients with EOC according to different prognosis and thus alternative therapeutic needs. We have recently published a validation study of a previously defined gene signature in 194 FIGO stage II-IV EOC patients, classifying the patients into two subclasses with significantly different prognosis. This analysis revealed the molecular subclassification and peritoneal carcinomatosis as the strongest independent prognostic factors for progression free and overall survival (HR 2.87 and HR 4.56, respectively). To better understand the differences characterizing these two subclasses, significance analysis of microarrays was performed whereby functional analysis of differentially expressed genes identified the focal adhesion pathway as one of the most deregulated pathways [4]. Interestingly, the central regulator, the focal adhesion kinase (FAK), was not among them. Thus, we hypothesized that not the different expression of the protein but the activation status (i.e. autophosphorylation) of FAK deregulates this pathway. This assumption and the fact that the inhibition of the autophosphorylation of FAK is discussed as therapeutic target in many cancer entities including EOC [5], leads to the aim of this study: To investigate FAK expression and pFAK abundance in serous EOC in regard to the molecular subclassification and patients’ outcome.

FAK promotes cell motility, invasion and proliferation in normal and cancer cells and was found to be overexpressed in a variety of cancer entities [6-8]. The high potential to disseminate within the peritoneal cavity and the ability of EOC cells to survive as single cells and multi-cellular spheroids in the ascitic fluid plays an important role in EOC [9]. Activation of FAK seems to be essential for anoikis resistance of epithelial cells without matrix contact. The involvement in anti-apoptotic functions and the ability to promote epithelial mesenchymal transition (EMT) has rendered FAK as a potential therapeutic target to inhibit tumor progression and metastasis [10-12].

Tyrosine 397 of the FAK protein is the main target of autophosphorylation, leading to the activation of FAK, and renders pFAK (Y397) as high-affinity partner for v-src sarcoma viral oncogene homolog (SRC) binding [13]. A number of inhibitors targeting FAK activation by blocking Y397-FAK phosphorylation have been investigated in in vivo and in vitro models of colon-, breast-, squamous-, and pancreatic cancer [14-16]. In vitro studies showed that the FAK inhibitor PND-1186 (proved by immunoblotting of pFAK-Y397) seems to trigger cell apoptosis and to inhibit cell motility in breast cancer cells [17]. In mouse models, PND-1186 inhibition of pFAK-Y397 resulted in reduced ovarian cancer tumor growth [17]. Recently, a phase I trial has investigated tolerability and antitumor activity of PF-00562271 in a heterogeneous population of 99 patients with advanced solid tumors [18]. Nevertheless, so far only one prognostic study using immunohistochemistry has focused on pFAK abundance and one study on FAK expression in human EOC tissues (n = 60 and n = 79, respectively) [19,20].

## Material and methods

### Study population

In the frame of an FP6 EU-project named OVCAD (http//http://www.ovcad.eu) we coordinated the development of a comprehensive and well described tumor bank and database of EOC patients. Within this project, samples from primary EOC were collected at the University clinics of Berlin, Hamburg, Innsbruck, Leuven, and Vienna (OVCAD-consortium) according to standardized operation procedures. Clinical and histopathological data as well as follow-up data were collected and cured by experienced clinicians. Patients presenting with benign ovarian diseases, low malignant potential ovarian cancer, clear-cell ovarian cancer, FIGO I stage EOC and patients with secondary malignant diseases were excluded [21]. The study protocol was approved by the Ethics Committees of the participating OVCAD partners (EK207/2003, ML2524, HEK190504, EK366, and EK260). All patients gave pre-operative written informed consent before enrollment in the study. Only patients undergoing debulking surgery and platinum–based chemotherapy were included to the OVCAD patient cohort. Overall survival (OS) was defined as the time interval between diagnosis and tumor associated death and progression free survival (PFS) as the time between cytoreductive surgery and disease progression or death. Overall observation time was the time interval between diagnosis and last contact, defined as death from the disease or last follow-up. Therapy response to chemotherapy was defined according to Chekerov et al. [21]. Patients were classified as non-responder if progression was diagnosed during treatment or recurrence within six months after end of first-line chemotherapy. Patients without recurrence, disease progression or death were censored at the time of last follow-up. Experienced gynecological oncologists and pathologists from the OVCAD consortium performed the clinical and histopathological evaluation and the evaluation of response to first-line treatment. As the impact of biomarkers on patients’ survival has been described to vary between histological subtypes [22] only patients with serous histology were included, resulting in a total of 179 serous EOC patients. Tumors were graded as well (1), moderately (2), or poorly differentiated (3).

### Immunostaining

**Figure 1:**
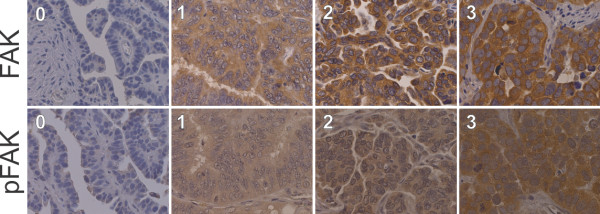
**Representative immunohistochemical examples of FAK and pFAK-Y397 staining.** FAK and pFAK staining intensities: 0, not stained at all; 1, low staining; 2, moderate staining; 3, strong staining. Pictures were taken using TissueFAXS, objective: ×40 (TissueGnostics, Austria).

Tissue micro arrays (TMAs) were constructed, whereby two 1 mm-diameter cores were obtained from each tumor sample. The immunohistochemistry procedure was performed essentially as described previously [23]. Antigen heat retrieval was performed using citrate buffer (Citra-BioGenex no. HK 087-5 K). The sections were incubated at 4°C overnight with primary antibodies (FAK, 1:200, monoclonal mouse IgG1, Millipore, catalog no. 05–182; pFAK, 1:150, polyclonal rabbit, abcam, catalog no. ab4803). As a positive control, breast cancer tissue sections and kidney tissue sections were used. For negative control breast and kidney sections were incubated in absence of the primary antibody. The specificity of the pFAK staining was shown by a peptide competition experiment, i.e. blocking of the primary antibody with the phosphorylated peptide mouse FAK (phospho Y397, abcam, catalog no. ab40145) in a molar excess ratio of 200-fold over night (Additional file 1: Figure S1). Samples were examined by two independent observers blinded for the clinical data, including a pathologist specialized in gynecology. FAK expression levels were determined using a scoring system based on staining intensity (0–3) and percentage of positive cells. pFAK scoring was performed as previously described (Fan and Shi, [19]), including staining intensity and percentage of positive cells. As both, FAK and pFAK staining were homogenous within the EOC tissue (Figure 1) percentage of positive cells did not differ significantly within the analyzed samples. The staining was grouped into FAK low and pFAK low (0–1; not stained at all and low expression) as well as FAK high and pFAK high (2–3; moderate to high expression).

Colocalisation of FAK and pFAK was determined by immunofluorescence staining of MCF7 and CaOV3 cells. The fluorescence labeled secondary antibodies, goat anti-rabbit (1:1,000; Invitrogen, AlexaFluor® 488 fragment of goat anti-rabbit IgG (H + L)) and goat anti-mouse (1:500; Invitrogen, AlexaFluor® 568 goat anti-mouse IgG1) were used besides DAPI for nuclear counterstaining. FAK staining intensity was designated as FAK *expression* and pFAK staining intensity as pFAK *abundance* throughout the paper, since phosphorylation is not a result of expression but a result of posttranslational modification.

### Microarray analysis

Microarray analysis of this patient cohort has been previously published by Pils et al. [4]. In the present study we tried to further elucidate differentially expressed genes between pFAK positive and pFAK negative samples. Microarray data were available from 141 samples [24]. The parameters pFAK (high, n = 51 vs. low, n = 90) and FAK (high, n = 132 vs. low, n = 9) were used for shrinkage and a non-parametric prior was calculated for the pFAK parameter (allowing non-unimodality). Bayesian False Discovery Rate (BFDR) values below 10% were considered as statistically significant [24]. Functional analysis of differentially expressed genes was performed with Database for Annotation, Visualization and Integrated Discovery (DAVID) v6.7 [25]. In addition a gene set enrichment analysis [26] using the Gene Set Database MSigDB v3.1 (http://www.broadinstitute.org/gsea/) was performed with the *romer* function [27] from the R-package limma v3.14.4 [28].

### Data analysis and statistics

Statistical analyses were performed using SPSS software version 19 (IBM Corporation, Armonk, New York, United States). Associations between FAK expression and pFAK abundance, and between these two factors and clinicopathological parameters were assessed by T-tests (age), Chi-square tests, and Fisher’s exact tests as appropriate. Results were adjusted for multiple testing by the Bonferroni-Holm method [29]. Impact on progression free survival (PFS) and overall survival (OS) was determined by univariate and multiple Cox proportional-Hazards regression model analyses. To assess the independent impact of factors not significant in the univariate Cox regression analyses, all factors were included in the multiple models according to suggestions from Harrell [30] and Sun et al. [31]. Impact on chemotherapy response was determined by univariate and multiple logistic regression models. In addition, the estimates of the impact of pFAK on overall survival (i.e. the multiple Cox regression model) corrected for the clinicopathologic parameters age, stage, grade, residual tumor load, and peritoneal carcinomatosis on overall survival was illustrated by survival curves. For this task all parameters were averaged and pFAK was used as stratifying variable.

## Results

### Study population

The characteristics of the 179 patients included in this study show a typical heterogeneous serous EOC population (Table 1 and 2). The clinicopathological characteristics are presented in Table 1 and 2 separately for the cohorts of pFAK-positive and -negative as well as FAK-positive and -negative patients, respectively. Mean age of the EOC patients at time of cytoreductive surgery was 57.6 years (SD ±12.6 years). The median observation period was 49 months (range: 1–69 months). Within the observation period, 82 patients died (45.8%) and 138 patients (77.1%) experienced tumor progression. A total of 43 patients (25%) did not respond to first-line chemotherapy.

**Table 1:** Characteristics of patients with serous epithelial ovarian cancer broken down by pFAK abundance

**n = 179 Characteristics**	**pFAK-low**	**pFAK-high**		
**Characteristics**	**n = 113 (%)**	**n = 66 (%)**	**p**	**Adjusted p**
**Age** [mean +/− SD]	56.6 [13.2]	59.5 [11.2]	0.135	
**FIGO**			**0.010**	0.050
II (n = 7)	6 (5.3)	1 (1.5)		
III (n = 142)	95 (84.1)	47 (71.2)		
IV (n = 30)	12 (6.7)	18 (27.3)		
**Grade** (1 missing)			**0.002**	**0.014**
Grade 1&2 (n = 49)	40 (35.7)	9 (13.6)		
Grade 3 (n = 129)	72 (64.3)	57 (86.4)		
**Residual tumor**			0.229	
no (n = 126)	76 (67.3)	50 (75.8)		
> 0 cm (n = 53)	37 (32.7)	16 (24.2)		
**Peritoneal carcinomatosis**			0.126	
no (n = 50)	36 (31.9)	14 (21.2)		
yes (n = 129)	77 (68.1)	52 (78.8)		
**Ascites**			0.546	
</= 500 ml (n = 106)	65 (57.5)	41 (62.1)		
> 500 ml (n = 73)	48 (42.5)	25 (37.9)		
**pNM**			**0.005**	**0.030**
N0 and M0 (n = 20)	18 (15.9)	2 (3.0)		
N1 and/or M1 (n = 111)	64 (56.6)	47 (71.2)		
NX or MX* (n = 48)	31 (27.4)	17 (25.8)		

P-values and adjusted p-values below 0.05 are shown in bold. *not included in statistics.

**Table 2:** Characteristics of patients with serous epithelial ovarian cancer broken down by FAK abundance

**n = 179**	**FAK-low**	**FAK-high**		
**Characteristics**	**n = 14 (%)**	**n = 165 (%)**	**p**	**Adjusted p**
**Age** [mean +/− SD]	56.1 [18.7]	57.8 [11.9]	0.645	
**FIGO**			0.150	
II (n = 7)	1 (7.1)	6 (3.6)		
III (n = 142)	13 (92.9)	129 (78.2)		
IV (n = 30)	0 (0)	30 (18.2)		
**Grade** (1 missing)			0.535	
Grade 1&2 (n = 49)	5 (35.7)	44 (26.8)		
Grade 3 (n = 129)	9 (64.3)	120 (73.2)		
**Residual tumor**			0.082	
no (n = 126)	7 (50.0)	119 (72.1)		
> 0 cm (n = 53)	7 (50.0)	46 (27.9)		
**Peritoneal carcinomatosis**			0.539	
no (n = 50)	5 (35.7)	45 (27.3)		
yes (n = 129)	9 (64.3)	120 (72.7)		
**Ascites**			0.869	
</= 500 ml (n = 106)	8 (57.1)	98 (59.4)		
> 500 ml (n = 73)	6 (42.9)	67 (40.6)		
**pNM**			**0.031**	0.217
N0 and M0 (n = 20)	4 (28.5)	16 (9.8)		
N1 and/or M1 (n = 111)	5 (35.7)	106 (64.6)		
NX or MX* (n = 48)	5 (35.7)	42 (25.6)		

P-values and adjusted p-values below 0.05 are shown in bold. *not included in statistics.

### Distribution of FAK expression and pFAK abundance in EOC

**Figure 2:**
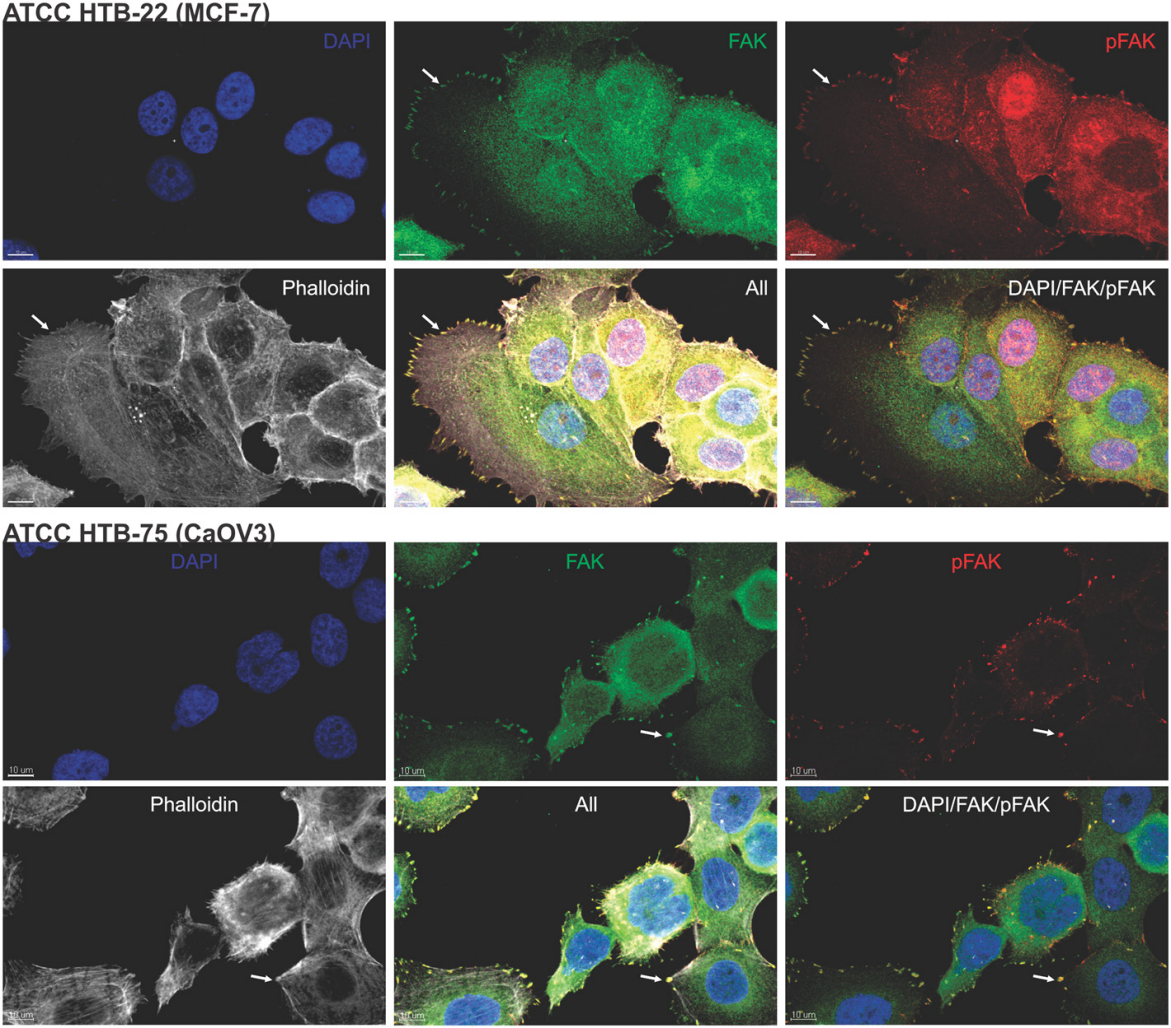
**Coexpression and distribution of FAK and pFAK-Y397 in MCF7 (breast) and CaOV3 (ovarian) cancer cell lines.** An intense FAK (green) and pFAK staining (red) can be observed within the membrane borders, interpreted as focal adhesions (white arrows). DAPI (blue) was used for nuclear counterstaining and Phalloidin (white) for staining of F-actin in the cytoplasm. (Pictures were taken with a confocal microscope LSM 700, Carl Zeiss AG, Germany).

For validation of the specificities of the FAK and the pFAK-Y397 antibodies immunofluorescence co-staining experiments were performed on MCF7 and CaOV3 cancer cell lines. As presented in Figure 2, both antibodies clearly stained focal adhesions, which proved the specificities of the used antibodies. A nuclear expression of pFAK (compared to only cytoplasmic expression of FAK) has been described previously by Murata et al. in colon cancer and breast cancer tissue. [32] In accordance with this data, staining of MCF7 cells also showed a nuclear staining of pFAK (Figure 2). However, in the ovarian cancer cell line CaOV3 and the immunohistochemistry stainings nuclear abundance could not be observed, as also described by Fan and Shi [19].

Immunohistochemistry of FAK and pFAK revealed a granular cytoplasmic immunostaining in serous EOC tumor tissue, typical for focal adhesion staining (Figure 1). High FAK staining was detected in a total of 92.2% (n = 165). pFAK was positive in a total of 87.2% and highly abundant in 36.9% (n = 66) of the samples. As expected, all the 66 samples showing high pFAK abundance also expressed FAK (p = 0.002). Comparing expression and abundance levels, a positive association between FAK and pFAK could be observed (p < 0.001).

### Correlation of pFAK abundance and FAK expression with clinicopathological parameters

pFAK abundance in grade 3 tumors was significantly higher than in grade 1&2 tumors (adjusted p = 0.014; Table 1). High pFAK abundance was also associated with the presence of either nodal positivity (N1) and/or distant metastasis (M1) (adjusted p = 0.030; Table 1). No significant differences were found for the clinicopathological parameters age, presence of residual tumor after debulking surgery, peritoneal carcinomatosis, and ascites (≤ vs > 500 ml). There was no association between FAK expression and the described clinicopathological parameters (adjusted p-values > 0.05; Table 2). The hypothesis, that pFAK abundance might be different between tumors of the previously described [4] molecular subclass 1 and molecular subclass 2 proved wrong. The subclasses were classified using a published 112 gene set [4]. Neither the FAK expression status correlated significantly with the molecular subclasses (p = 0.910), which is in accordance with previous data [4], nor the pFAK-Y397 abundance (p = 0.108), which was the initial hypothesis of this study.

### Survival analyses

Table 3 shows the impact of pFAK abundance and FAK expression on OS and PFS in the subgroup of FIGO III/IV serous EOC (n = 172), together with various clinicopathological parameters considered as potential prognostic factors. In univariate analyses, age (in decades) (hazard ratio [HR] 1.48; p < 0.001), FIGO stage (HR 2.48; p < 0.001), residual tumor (HR 1.77; p < 0.001), peritoneal carcinomatosis (HR 3.11; p < 0.001) and the molecular subclassification (HR 2.51; p = 0.001) affected OS. Likewise, PFS was negatively affected by higher age, FIGO IV stage, residual tumor, peritoneal carcinomatosis, and molecular subclass.

**Table 3:** Multiple Cox regression analyses for progression-free and overall survival, both for the proportion of explained variations of clinicopathologic parameters and FAK as well as pFAK for late stage serous ovarian cancer patients (n = 172)

**A. Overall survival**				
	**Univariate, n = 172**		**Multiple, n = 140**	
**Characteristics**	**HR (CI 95%)**	**p**	**HR (CI 95%)**	**p**
**Age (per decade)**	**1.48 (1.22-1.79)**	**<0.001**	**1.35 (1.08-1.69)**	**0.010**
**FIGO (IV vs III)**	**2.48 (1.51-4.06)**	**<0.001**	**3.35 (1.84-6.09)**	**<0.001**
Grade (3 vs 1,2)	1.56 (0.91-2.68)	0.105	1.64 (0.83-3.24)	0.155
Residual tumor (yes vs no)	**1.77 (1.12-2.78)**	**0.014**	1.33 (0.78-2.24)	0.292
**Peritoneal carcinomatosis (yes vs no)**	**3.11 (1.64-5.90)**	**<0.001**	**3.18 (1.43-7.11)**	**0.005**
**Yoshihara subclassification (subclass 2 vs 1)**	2.51 (1.49-4.24)*	**0.001**	**2.23 (1.29-3.87)**	**0.004**
FAK (high vs low)	0.85 (0.41-1.77)	0.671	0.91 (0.37-2.24)	0.830
**pFAK (high vs low)**	0.80 (0.50-1.27)	0.343	**0.54 (0.31-0.96)**	**0.034**
**B. Progression free survival**				
	**Univariate, n = 172**		**Multiple, n = 140**	
**Characteristics**	**HR (CI 95%)**	**p**	**HR (CI 95%)**	**p**
**Age (per decade)**	**1.25 (1.08-1.44)**	**0.003**	**1.19 (1.01-1.41)**	**0.042**
**FIGO (IV vs III)**	**2.81 (1.83-4.31)**	**<0.001**	**3.04 (1.83-5.06)**	**<0.001**
Grade (3 vs 1,2)	1.32 (0.89-1.97)	0.171	1.01 (0.61-1.66)	0.971
Residual tumor (yes vs no)	**1.94 (1.35-2.81)**	**<0.001**	1.40 (0.91-2.17)	0.127
**Peritoneal carcinomatosis (yes vs no)**	**2.77 (1.78-4.32)**	**<0.001**	**3.13 (1.80-5.46)**	**<0.001**
Yoshihara subclassification (subclass 2 vs 1)	1.61 (1.09-2.36)**	**0.016**	1.36 (0.90-2.04)	0.142
FAK (high vs low)	0.87 (0.48-1.58)	0.654	0.89 (0.41-1.95)	0.776
pFAK (high vs low)	1.15 (0.81-1.63)	0.425	1.02 (0.66-1.57)	0.927

P-values and adjusted p-values below 0.05 are shown in bold. *n = 141 (due to the limited availability of microarray data).

**Figure 3:**
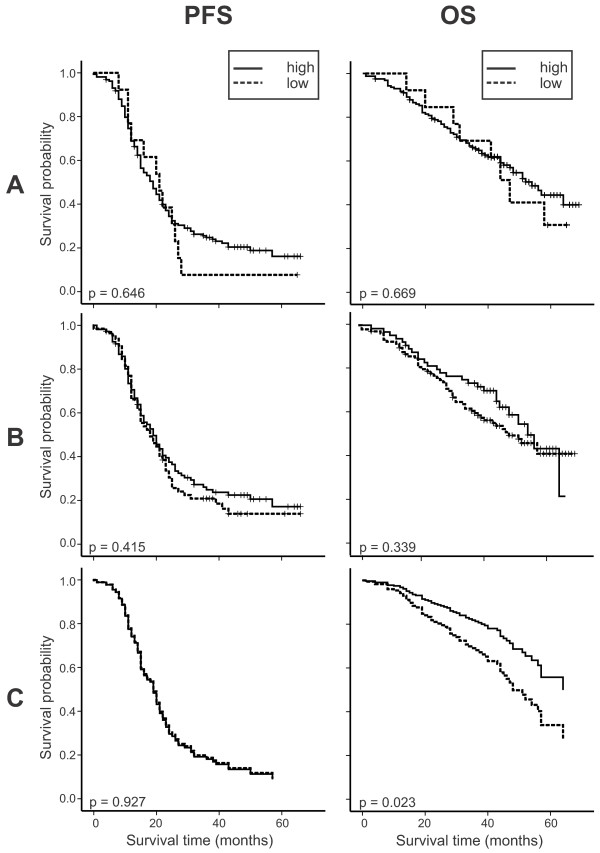
**Kaplan–Meier estimates of the univariate impact of FAK (A) and (B) pFAK-Y397 and survival curves of the final Cox regression model (C), corrected for the clinicopathologic parameters age, stage, grade, residual tumor load, peritoneal carcinomatosis, and molecular subclass (i.e. all of these factors were averaged and pFAK was used as stratifying variable) on progression free and overall survival.** In contrast to Kaplan-Meier plots, where it is common to indicate censored observations, Figure 1C shows survival estimates derived from the Cox model, as explained in the statistical analysis section. In this plot it is uncommon to graphically show censored observations. pFAK low, n = 90 versus pFAK high, n = 50 patients.

In addition, the impact of FAK and pFAK together with the molecular subclassification on FIGO III and IV serous tumors was analyzed by multiple Cox regression. In the final model, pFAK (HR 0.54; p = 0.034) and molecular subclass (HR 2.23; p = 0.004) showed a significant independent impact on OS besides age, FIGO stage, and the presence of peritoneal carcinomatosis. Although residual disease is known as an important clinical prognostic factor, it was not independently prognostic in the multiple analysis, because it was outperformed by the other factors in the model - probably by the very closely linked factor peritoneal carcinomatosis. For PFS, the parameters age, stage, and peritoneal carcinomatosis showed a significant independent impact but neither FAK, pFAK nor the molecular subclass did (Table 3). Figure 3 shows the univariate impact of pFAK on PFS and OS and the impact of pFAK, corrected for the relevant clinicopathologic parameters, on prognostication of PFS and OS as estimated survival curves (Figure 3C).

The predictive value of FAK and pFAK abundance, i.e. influencing response to first-line chemotherapy, was estimated by multiple logistic regression analyses. The risk to be a non-responder was neither significantly influenced by FAK expression, nor by pFAK abundance (HR 1.13, 95% CI [0.29-4.31], p = 0.858; HR 0.73, 95% CI [0.35-1.51], p = 0.729, respectively).

### Gene expression analysis

Using an empirical Bayesian approach for differential gene expression analysis between pFAK high and pFAK low abundant samples, 780 ProbeIDs were found to be statistically (BFDR < 10%) significantly differentially expressed. An over-representation analysis (602 of the 780 ProbeIDs were annotated in DAVID) revealed the annotation clusters “ribosome” and “RNA processing” as over-represented (Additional file 2: Figure S2; Enrichment Scores 5.26 and 2.34, respectively) from the differentially expressed genes and the KEGG pathway “Ribosome” as the most over-represented single pathway (Additional file 3: Figure S3; Benjamini-Hochberg corrected, p = 0.00022). A sensitive Gene Set Enrichment Analysis using 6,769 gene sets and the *romer*-function from the limma package revealed 227 down-regulated, 300 mixed (partly down- and partly up-regulated), and 414 up-regulated gene sets as significantly deregulated in pFAK high tumors (FDR < 5%; totaling in 862 gene sets; Additional file 4: Table S1; overlap shown in Additional file 5: Figure S4). Among the up-regulated gene sets were the “Creighton AKT1 signaling via mTOR” [33], the “mRNA-binding” and the “microtubule associated complex” gene set and among the mixed gene set was the “cell adhesion molecules” gene set. For FAK no such analysis was performed mainly because there were only nine FAK negative samples compared to 132 FAK positive samples, which renders a very unbalanced, thus underpowered, design.

## Discussion

Several approaches to inhibit FAK kinase activity in anticancer therapy have been published but little is known about the role and prognostic value of FAK and phosphorylated FAK (pFAK) in EOC. Moreover, in a previous study we found that the focal adhesion pathway was the mostly deregulated pathway in two distinct molecular subclasses of EOC with different prognosis [4]. As FAK was not differentially expressed, we hypothesized that phosphorylation of FAK could have accounted for the difference between the two subclasses. Therefore, we found it important to determine the abundance and prognostic impact of FAK and especially pFAK-Y397 in a large and well-described stage II-IV serous EOC patient cohort.

Addressing the prognostic impact of FAK and pFAK-Y397, we did not find an association between FAK expression and survival, concluding that tumor expression of FAK alone is of limited prognostic value in advanced stage EOC. However, pFAK abundance was associated with significantly better OS in multiple analysis in this cohort of FIGO III/IV stage serous EOC patients (HR 0.54 (0.31-0.96), p = 0.034). This finding is surprising in view of previous in vitro data describing that an inhibition of FAK phosphorylation leads to decreased invasion and migration of 222 and SKOV3 cancer cells [20]. In mouse models, an inhibition of pFAK-Y397 resulted in reduced ovarian cancer tumor growth [17].

Reports describing pFAK abundance in human serous EOC are scarce. To our knowledge, only one previous study [19] investigated the prognostic impact of pFAK in EOC. Fan and Shi showed, that pFAK alone was no independent risk factor for prognosis in EOC but DLC1 negative combined with pFAK positive patients showed a shorter OS [19]. An explanation for these results might be the population itself. Fan and Shi included 60 advanced stage EOC patients in their survival analysis (mean follow-up 36 months) while we analyzed a larger population of 140 advanced stage EOC patients of only serous histology (mean follow-up 49 months). As the majority of associations vary significantly between the histological subtypes, studies seeking prognostic biomarkers in ovarian cancer should be adequately accounted for histotype [22]. Additionally the factors residual tumor load, FIGO III vs IV, and age, commonly used as correcting factors in multiple analysis, are missing in this study.

Disagreeing to data indicating pro-tumorigenic and pro-metastatic effects in vitro and in vivo (mouse) and a negative impact of high FAK expression on survival in other tumor entities, we showed that FAK had no influence on prognosis in advanced stage serous EOC. In contrast, high pFAK abundance had an independent positive impact on OS, although we also find a positive correlation of high pFAK abundance with lymph-node and/or distant metastasis. However, correcting for the most relevant clinicopathologic factors, high pFAK abundance is an independent positive risk factor. As described by Kohn et al. [34] EOC does not follow the classical rules observed in other cancer entities: cancer spread within the peritoneal cavity seems to be an early event and of much more relevance than lymphovascular dissemination resulting in distant metastasis. According to clinical data, distant metastasis does not per se influence prognosis, therefore oncogenes involved in the process of distant metastasizing seem to play a minor role in EOC. As previously published, similar contradictory results were described for CCNE1 [35] and TRAP1 [23], both pro-tumorigenic in in vitro analyses but both independent positive predictors for OS in EOC. EOC patients are usually diagnosed with disseminated intraperitoneal tumor spread. This indicates that especially the specific microenvironment (e.g. highly anaerobic) in the peritoneal cavity has an important influence on recurrence and survival in this tumor entity.

Comparing FAK expression in human EOC tissues (n = 5) and normal ovarian tissues (n = 3), an overexpression of FAK was seen in EOC cells [36]. Addressing immunohistochemically determined FAK expression in EOC, Sood et al. described an overexpression of FAK in the majority of EOC patients (80%, n = 79) [20], comparable to our high FAK expression rate of 92.2% (n = 179). The increased expression and activation of FAK in EOC, consistent with its role in invasion, cell migration, angiogenesis and proliferation highlights the clinical importance of this tyrosine kinase.

To our knowledge, only one other study including 76 EOC patients of serous and non-serous histology, analyzed pFAK abundance in EOC tissue [19] whereby the pFAK positive rate was 67.1%, compared to our pFAK positive rate of 87.2% (staining intensity > 0). We found a significant association of high pFAK abundance and high tumor grade and an association of high pFAK abundance with the presence of either nodal positivity and/or distant metastasis. In accordance to our findings, an association between nodal positivity and pFAK abundance was also seen by Fan and Shi [19]. The ability of tumor cells to survive in different environments seems to be influenced by FAK signaling. Cell detachment, migration, proliferation and invasion are crucial components of this complex process of intraperitoneal tumor spread and metastasis. The activation and phosphorylation of FAK is stimulated by integrins and involved in cellular interactions with the extracellular matrix. Phosphorylation of FAK at Y397 is critical for its scaffolding function and its role in cell motility [37], it has been shown to promote transendothelial migration of breast cancer cells [38] and to initiate a cascade of phosphorylation events and a variety of cellular functions involved in cell migration and metastasis [37-39]. The correlation with lymph node and distant metastasis gives additional clinical information besides already published in vivo [40] and in vitro [41] data on the clinical importance of the Y397 FAK phosphorylation site.

As shown by functional analysis of the differences in the transcriptome between tissues of molecular subclass 1 and molecular subclass 2, described by Pils et al. [4], the focal adhesion pathway was the most deregulated pathway at all. Contradictory to our main hypothesis, that the two molecular subclasses might differ in levels of activated pFAK-Y397, abundance of pFAK was not significantly different in the two subclasses. The previously described prognostic impact of the molecular subclassification on OS [4] remained high and significant also including FAK and pFAK in the multiple Cox regression analysis (HR 2.23 (1.29-3.87), p = 0.004), indicating an independent influence of the molecular subclassification and pFAK abundance on OS.

Gene expression analysis comparing pFAK high and pFAK low tissues revealed that the expression of ribosomal proteins was highly deregulated, which was somewhat surprising. Recently, Kim et al. showed a clear link between the mTOR pathway and pFAK via the S6 kinase 1 (S6K1) in esophageal squamous cell carcinoma [42]. mTOR regulates ribosomal gene expression via S6K1, i.e. high S6K1 expression leads to high S6 (small ribosomal protein S6) expression and subsequently to larger cell sizes [43] and cell growth but also to induced phosphorylation of tyr-397 of FAK. Kim et al. showed that lowering the levels of S6K1 and S6 led to impairment of focal adhesion formation, which was paralleled by a reduction in phosphorylation of FAK and paxilin controlling. Correspondingly, our data showed that high pFAK abundance correlated with higher expression of ribosomal proteins in general. The PI3K/AKT/mTOR pathway is a well-known pathway involved in ovarian cancer carcinogenesis and mTOR as a drug target (e.g.: Rapamycin, Everolimus, and Temsirolimus) is being investigated in clinical trials [44,45]. Therefore, it is interesting that the PI3K/AKT/mTOR pathway seems to be connected to the FAK-pFAK axis via S6K1 [46] in EOC (a pathway showing the connection between mTOR and FAK is depicted in Additional file 6: Figure S5, indicating also the expression changes of the key player).

This study showed that the role of FAK and pFAK-Y397 in high stage serous EOC patients is not as straight forward as expected from in vitro and mouse models and that the therapeutic effect of blocking Y397-FAK phosphorylation could be at least ambiguous: on the one hand high pFAK abundance was associated with increased lymph node and distant metastases (known to be of minor importance in ovarian cancer prognosis) but on the other hand patients with high pFAK abundance showed better prognosis if corrected for all histopathologic parameters.

## Competing interests

The authors have no competing interests to declare.

## Authors’ contributions

SA, KA, and ABH performed immunohistochemistry and immunofluorescence stainings, analyzed the data and drafted the manuscript; CD, JS, IB, SM, SL, IV, and CG provided clinical samples and data; SA and RH reviewed immunohistochemistry staining and clinicopathologic information, DCT managed sample and data collection, RZ supervised sample and data collection; and DP planed together with SA, KA, and ABH the study, supervised statistical analysis, analyzed microarray data, and drafted the manuscript. All authors read and approved the final manuscript.

## Supplementary Material

Additional file 1: Figure S1 Peptide competition experiment, showing the specificity of the pFAK antibody on kidney positive control tissue sections (NC, negative control omitting primary antibody; Ab + peptide, pre-incubating primary antibody with 200-fold molar excess of phosphorylated FAK peptide over night; pFAK, positive staining). Click here for file

Additional file 2: Figure S2 Significantly enriched DAVID annotation clusters. DAVID analysis using the 780 significantly differentially expressed ProbeIDs (thereof 602 annotated in DAVID) revealed the annotation clusters “ribosome” and “RNA processing” as over-represented. Click here for file

Additional file 3: Figure S3 The significantly overrepresented KEGG pathway “ribosome” with the significantly deregulated genes labeled by asterisks. Click here for file

Additional file 4: Table S1 List of the 227 down-regulated, 300 mixed (partly down- and partly up-regulated), and 414 up-regulated gene sets (FDR < 5%; totaling in 862 gene sets) found as significantly deregulated by pFAK abundance (cf. Additonal file 3: Figure S3). Click here for file

Additional file 5: Figure S4 Venn-plot of the overlap of the 227 down-regulated, 300 mixed (partly down- and partly up-regulated), and 414 up-regulated gene sets (FDR < 5%; totaling in 862 gene sets) found as significantly deregulated by pFAK abundance (cf. Additional file 4: Table S1). Click here for file

Additional file 6: Figure S5 Pathway analysis showing the axis mTOR (AKT)-S6K1/RPS6KB1-FAK/PTK2 with gene expression values averaged over all pFAK low (left) and pFAK high (right) samples. (The pathway was built with GeneSpring 11.5.1 using the mTOR KEGG-pathway genes, RPS6KB1, and PTK2 as seeding proteins: Expand Interactions; Relations score > = 9; Relation types chosen: Expression, Regulation, Binding, Protein Modification; Entity local connectivity > = 9; Entity types chosen: Enzyme, Protein; Limit results by Local to Global Connectivity Ratio; Limit results to: 8 new entities). Click here for file
