# Supplementary figures and images for: Ambivalent role of pFAK-Y397 in serous ovarian cancer-a study of the OVCAD consortium
Source: Mol Cancer. 2014 Mar 21;13:67. doi: 10.1186/1476-4598-13-67 (PMC3998046; doi:10.1186/1476-4598-13-67)

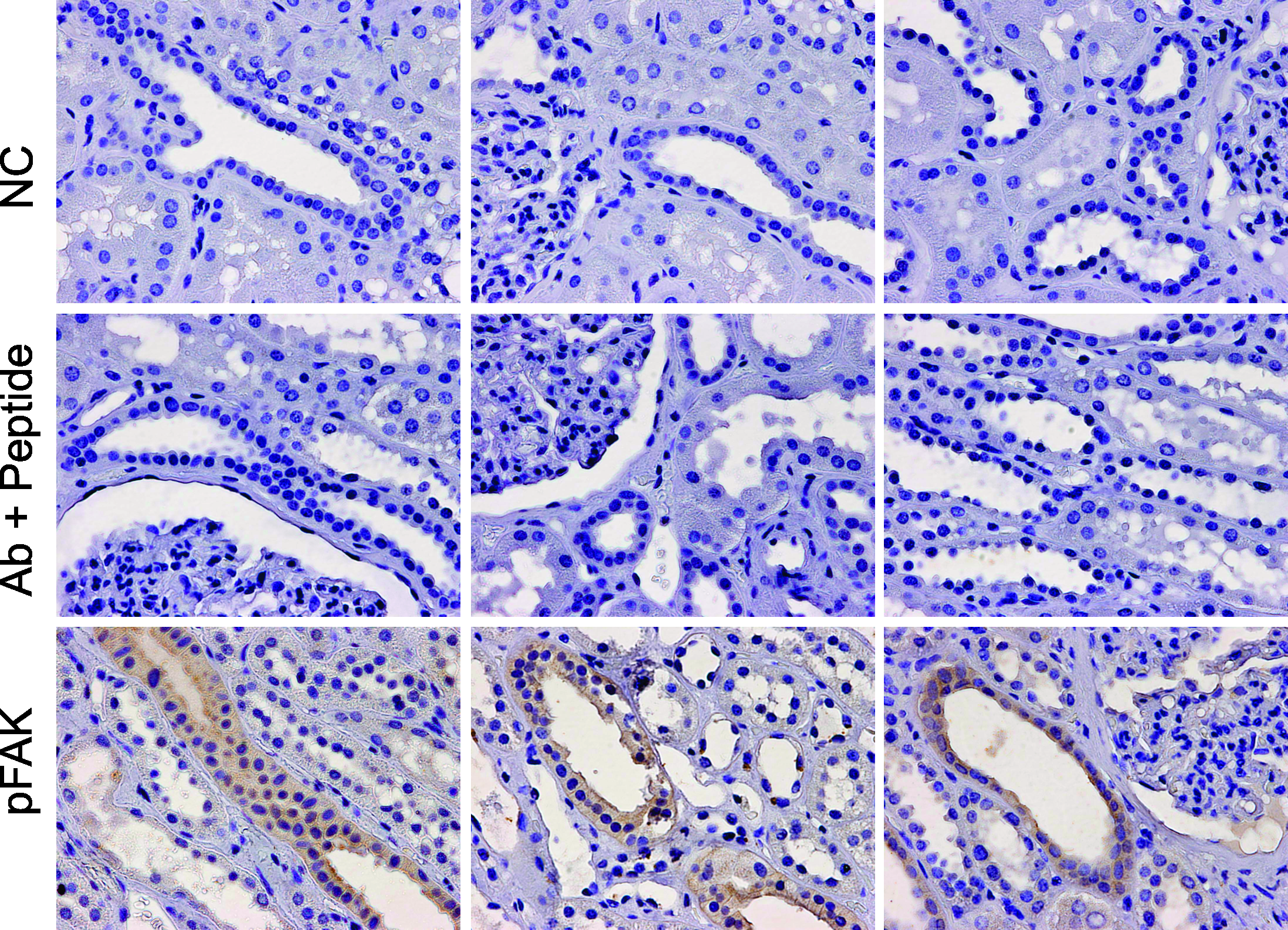

Supplement: Additional file 1: Figure S1 — Peptide competition experiment, showing the specificity of the pFAK antibody on kidney positive control tissue sections (NC, negative control omitting primary antibody; Ab + peptide, pre-incubating primary antibody with 200-fold molar excess of phosphorylated FAK peptide over night; pFAK, positive staining). [file 1476-4598-13-67-S1.tiff]

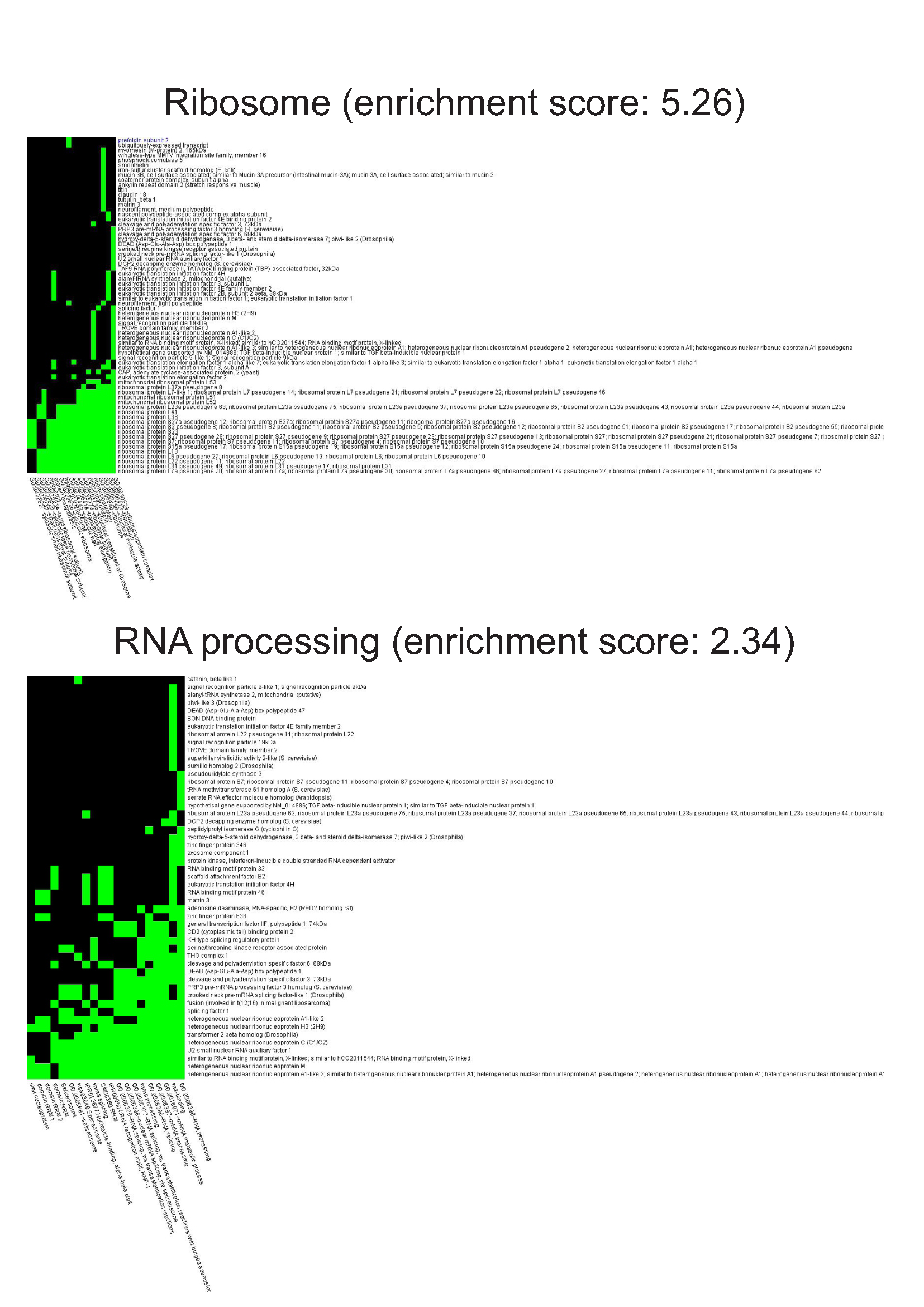

Supplement: Additional file 2: Figure S2 — Significantly enriched DAVID annotation clusters. DAVID analysis using the 780 significantly differentially expressed ProbeIDs (thereof 602 annotated in DAVID) revealed the annotation clusters “ribosome” and “RNA processing” as over-represented. [file 1476-4598-13-67-S2.tiff]

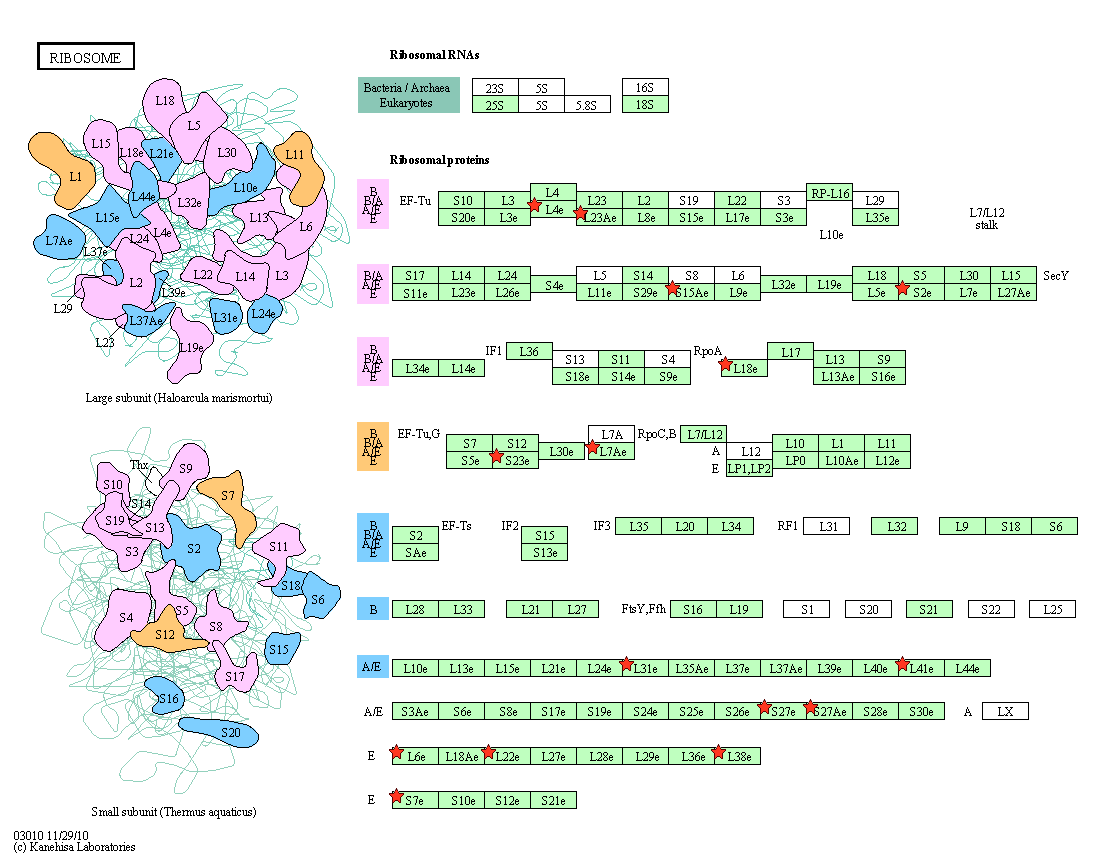

Supplement: Additional file 3: Figure S3 — The significantly overrepresented KEGG pathway “ribosome” with the significantly deregulated genes labeled by asterisks. [file 1476-4598-13-67-S3.tiff]

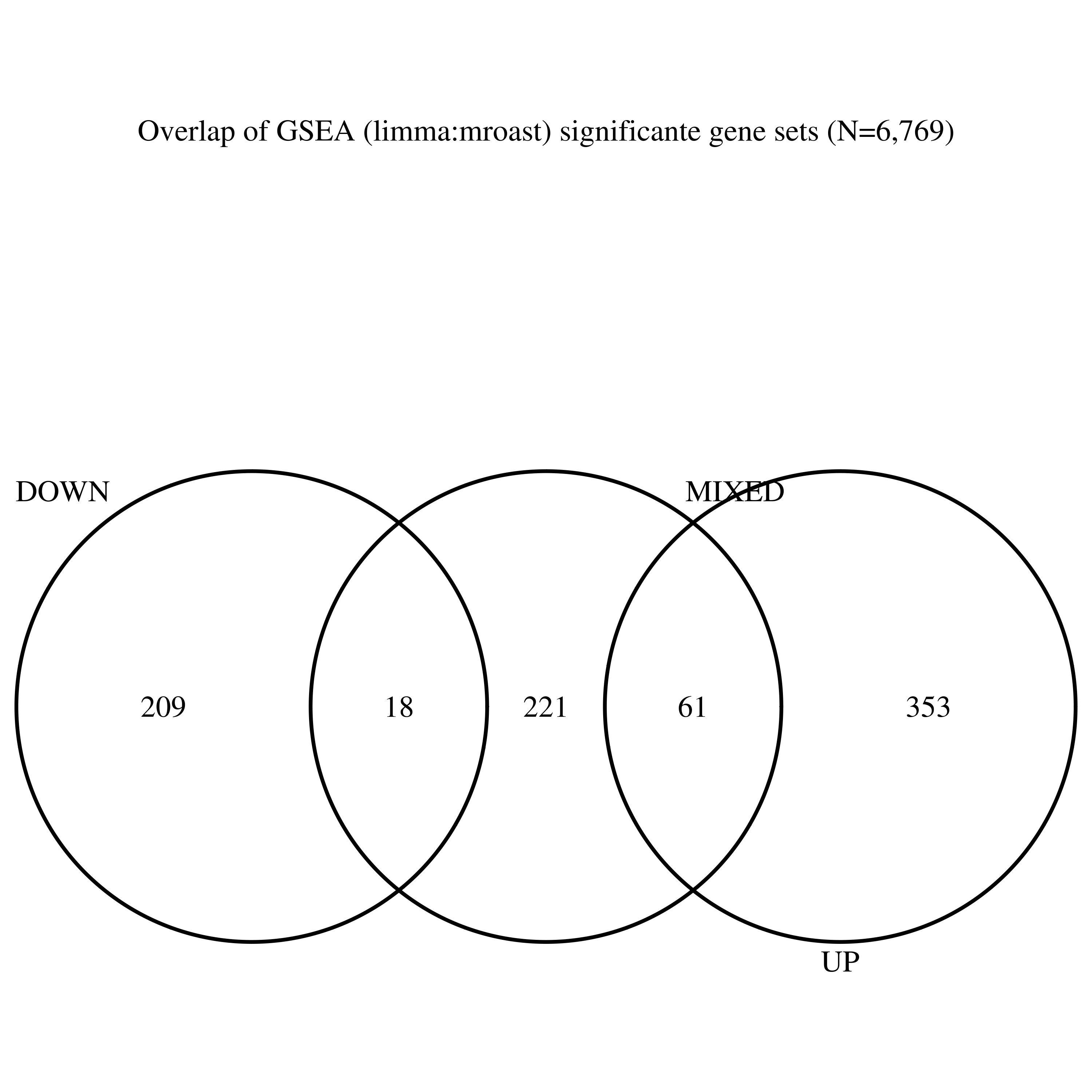

Supplement: Additional file 5: Figure S4 — Venn-plot of the overlap of the 227 down-regulated, 300 mixed (partly down- and partly up-regulated), and 414 up-regulated gene sets (FDR < 5%; totaling in 862 gene sets) found as significantly deregulated by pFAK abundance (cf. Additional file 4: Table S1). [file 1476-4598-13-67-S5.tiff]

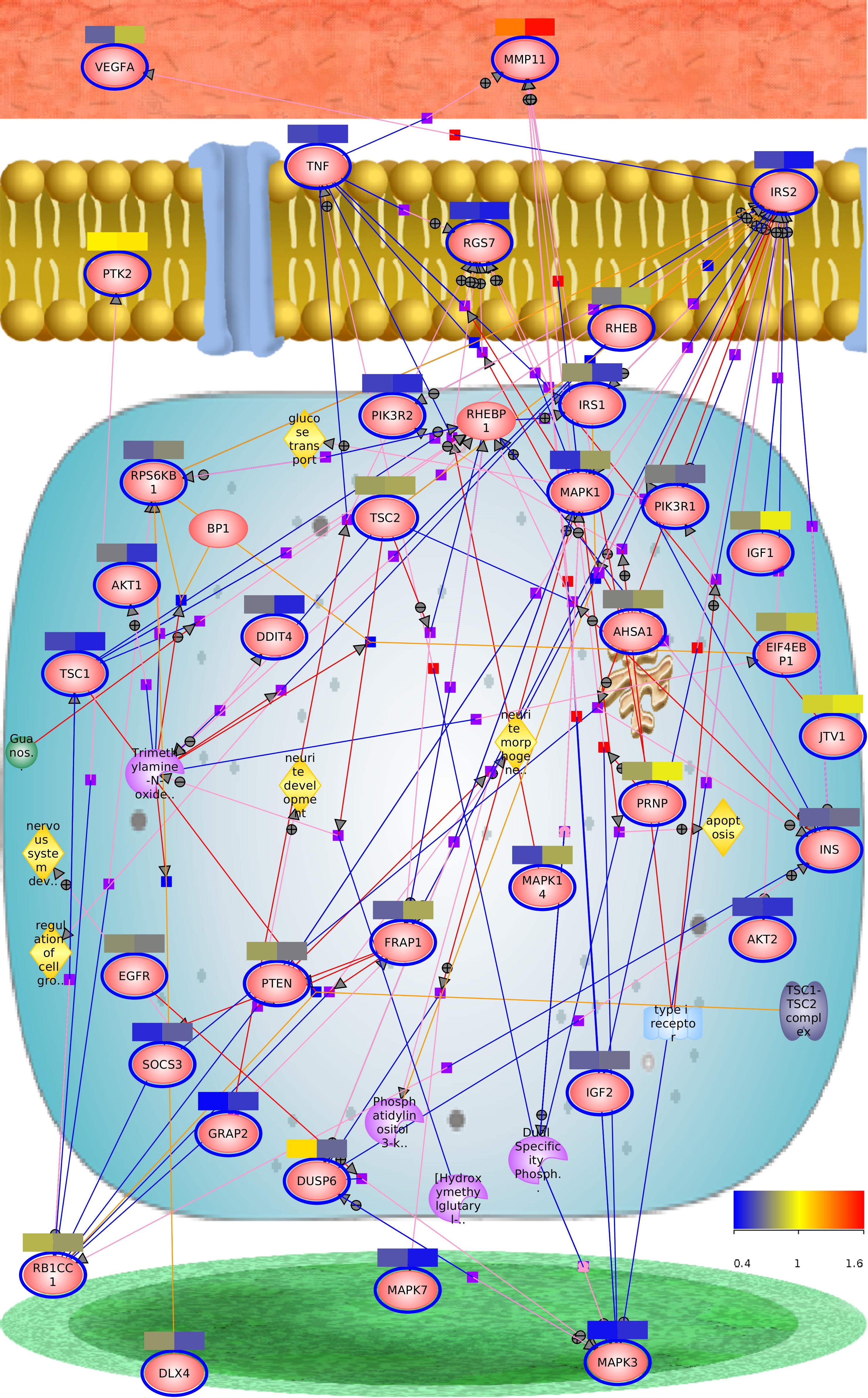

Supplement: Additional file 6: Figure S5 — Pathway analysis showing the axis mTOR (AKT)-S6K1/RPS6KB1-FAK/PTK2 with gene expression values averaged over all pFAK low (left) and pFAK high (right) samples. (The pathway was built with GeneSpring 11.5.1 using the mTOR KEGG-pathway genes, RPS6KB1, and PTK2 as seeding proteins: Expand Interactions; Relations score > = 9; Relation types chosen: Expression, Regulation, Binding, Protein Modification; Entity local connectivity > = 9; Entity types chosen: Enzyme, Protein; Limit results by Local to Global Connectivity Ratio; Limit results to: 8 new entities). [file 1476-4598-13-67-S6.jpeg]
